# Comparison of Chosen Physical Fitness Characteristics of Turkish Professional Basketball Players by Division and Playing Position

**DOI:** 10.2478/v10078-011-0077-y

**Published:** 2011-12-25

**Authors:** Yusuf Köklü, Utku Alemdaroğlu, Fatma Ünver Koçak, A. Emre Erol, Gülin Fındıkoğlu

**Affiliations:** 1 Pamukkale University, School of Sport Sciences and Technology, Denizli, Turkey; 2 Gazi University, School of Physical Education and Sports, Ankara, Turkey; 3 Pamukkale University, Physical Therapy and Rehabilitation, Denizli, Turkey

**Keywords:** Body composition, basketball players, isokinetic leg strength, maximal oxygen uptake, agility, speed

## Abstract

The purpose of the present study was to compare chosen physical fitness characteristics of Turkish professional basketball players in different divisions (first and second division) and playing positions. Forty-five professional male basketball players (14 guards, 15 forwards, 16 centers) participated in this study voluntarily. For each player, anthropometric measurements were performed, as well as a multi-stage 20 m shuttle run, isokinetic leg strength, squat jump (SJ), countermovement jump (CMJ), 10–30 meter single-sprint and T-drill agility tests. The differences in terms of division were evaluated by independent t-test and the differences by playing position were evaluated by one-way ANOVA with Post Hoc Tukey test. First division players’ CMJ measurements were significantly higher than those of second division players’ (p≤0.05), whereas second division players’ 10 m sprint times were significantly better than those of first division players’ (p≤0.05). In addition, forwards and centers were significantly taller than guards. Centers were significantly heavier and their T-drill test performances were inferior to those of forwards and guards (p≤0.05). Moreover, guards had a significantly higher maximal oxygen uptake (VO_2_ max) than centers. Guards and forwards showed significantly better performance in the 10 and 30 m sprint tests than centers (p≤0.05). Forwards and centers had significantly better left leg flexor strength at 180°.s^−1^(p≤0.05). In conclusion, the findings of the present study indicated that physical performance of professional basketball players differed among guards, forwards and centers, whereas there were not significant differences between first and second division players. According to the present study, court positions have different demands and physical attributes which are specific to each playing position in professional basketball players. Therefore, these results suggest that coaches should tailor fitness programs according to specific positions on the court.

## Introduction

Basketball is a predominantly anaerobic sport discipline, where most of the energy demands for high intensity activities such as, starts, stops, and changes of direction, jumps, shots, blocks and rebounds come from the creatine phosphate system (CP) (Delextrat and Cohen, 2009; Meckell et al., 2009; Metaxas et al., 2009). Anaerobic glycolysis with the production of lactate as a metabolic by-product is incorporated less often in game situations and occurs only when a high intensity activity lasts for 10 to 30 s and energy has to be derived from muscle glycogen stores. Such situations appear during a full court press or during quick transition from defense to offence and vice-versa. Aerobic metabolism dominates during breaks (time outs or substitutions) and low intensity activities such as standing, walking, ball inbounding or free throw shooting (Drinkwater et al., 2008). A high level of aerobic fitness allows for a quick recovery between bouts of high intensity activities, since muscle CP stores may be replenished within 30–40s if the player’s level of aerobic capacity is high (Castagna et al., 2008; Tomlin et al., 2001).

During a basketball game, professional players cover about 3500–5000 m (Janeira and Maia, 1998). Each player performs close to 1000 brief activities which change approximately every 2 seconds. Time motion analyses have shown that these short activities are performed with a different frequency according to the player’s position (Abdelkerim et al., 2007). Guards are involved in high intensity activities such as sprints and dribbles more often than forwards and centers; centers carry out more jumps (for offensive and defensive rebounds), also walking and standing are more frequent than in forwards and guards. Forwards take more shots and do more walking and standing than guards and centers (Abdelkerim et al., 2007; Drinkwater et al., 2008).

Several studies have examined some physical fitness characteristics of basketball players in different divisions and playing positions. For example Ostojic et al. (2006) showed that a strong relationship exists between body composition, aerobic fitness, anaerobic power, and position roles in elite basketball. Sallet et al. (2005) compared physiological characteristics of first and second division French professional basketball players, and related them to playing position and division level. Sallet et al.’s results demonstrated that selecting players for high level competition not only implies specific morphological characteristics, but also depends on particular physiological and technical profiles. Abdelkrim et al. (2010) compared the physical attributes of elite men’s basketball players according to age and specific individual position on the court. Abdelkrim et al. indicated the existence of age and court position differences in fitness performance in men’s basketball. However, to our knowledge, there is little information available concerning the physical fitness characteristics of professional European basketball players. Therefore, the evaluation of professional European players’ physical fitness characteristics must be done according to different divisions and playing positions. Based on this assumption, the purpose of the present study was the comparison of Turkish professional basketball players’ body composition, isokinetic leg strength, endurance, speed, vertical jump and agility performances by division and a playing position.

## Methods

### Subjects

Twenty-two Turkish first division basketball players (average age 24.0 ± 3.8 years; body height 197.9 ± 8.0 cm; body mass 98.4 ± 12.3 kg) and twenty-three Turkish second division basketball players (average age 22.7 ± 4.0 years; body height 195.7 ± 7.4 cm; body mass 94.7± 14.4 kg) participated in this study voluntarily. The subjects were fully informed about applied procedures, the experimental risk and written informed consent was obtained from all of them.

### Experimental Approach to the Problem

On the first day, the players participated in anthropometric measurements (body height, body mass, percentage of body fat) followed by a squat jump, countermovement jump and Multistage 20-meter shuttle run tests on the third day. Then, on the fifth day, 10 and 30 m sprints and the T-drill test were carried out. Isokinetic leg strength tests were conducted on the seventh day.

### Anthropometric Measurements

Subjects reported to the laboratory at 8 a.m. On entering the laboratory, body height (cm), body mass (kg), and percentage of body fat (%) measurements were taken for each subject. The body height of the basketball players was measured using a stadiometer with the accuracy to 1 cm (SECA, Germany), while electronic scales (Tanita BC 418, Japon) accurate to 0.1 kg were used for body mass and percentage of body fat measurements (Lohman et al., 1988).

### Multi-Stage 20-m Shuttle Run Test

The subjects’ maximal oxygen uptake (VO_2_max) was indirectly obtained using a multistage 20-m shuttle run test (Leger et al., 1988). This consisted of shuttle running between two parallel lines set 20 m apart, running speed cues being indicated by signals emitted from a commercially available pre-recorded audiocassette tape. The audiocassette tape ensured that subjects started running at initial speed of 8.5 km × h^−1^ and that running speed increased by 0.5 km × h^−1^ each minute. This increase in running speed is described as a change in test level. The speed of the cassette player was checked for accuracy in accordance with the manufacturer’s instructions before each application. All subjects performed a 10 min warm up that included prescribed jogging and stretching. Test results for each subject were expressed as a predicted VO_2_max obtained by cross-referencing the final level and shuttle number (completed) at which the subject volitionally exhausted with that of the VO_2_max table provided in the instruction booklet accompanying the multi-stage 20-m shuttle run test. Only fully completed 20-m shuttle runs were considered.

### Isokinetic Leg Strength:

Before the isokinetic test, subjects performed a 5-min warm up on a bicycle ergometer. Measurements were taken using an Isomed 2000 (Ferstl, Germany) isokinetic dynamometer. The test was performed in a seated position; stabilization straps were secured across the trunk, waist, and distal femur of the tested leg. The leg extensor and leg flexor muscle of each leg were concentrically measured at 60° × s^−1^ (10 repetitions) and 180° × s^−1^ (10 repetitions). Verbal encouragement was given to the subjects during the measurement. Before starting the test, subjects were allowed 5 trials.

### Vertical Jump Measurements

Vertical jump performance was assessed using a portable force platform (Newtest, Finland). Players performed countermovement (CMJ) and squat jumps (SJ) according to the protocol described by Bosco et al. (1983). Before testing, the players performed self-administered submaximal CMJs and SJ (2–3 repetitions) to get familiar with the testing procedures. They were asked to keep their hands on their hips to prevent any influence of arm movements on the vertical jumps and to avoid coordination as a confounding variable in the assessment of leg extensors (Bosco et al. 1995). Each subject performed 3 maximal CMJs and SJs, with approximately 2 minutes recovery in between. Players were asked to jump as high as possible; the best score was recorded in centimeters (Bosco et al.. 1995).

### 10 and 30 m Sprint Test

The subjects performed 2 maximal 30 m sprints (with 10 m split times also recorded) on the basketball court. There was a recovery period of 3 minutes between the 30 m sprints. Prior to each sprint, players performed a thorough warm-up consisting of 10 minutes of jogging at 60–70% of HRmax and then 5 minutes of exercises involving fast leg movements (e.g. skipping, cariocas) over short distances of 5 to 10 m and 3–5 single 15 m shuttle sprints with 2 minutes of passive recovery. Time was measured using an electronic timing system (Prosport TMR ESC 2100, Tumer Engineering, Ankara, Turkey).

### T-Drill Agility Test:

**Figure 1:**
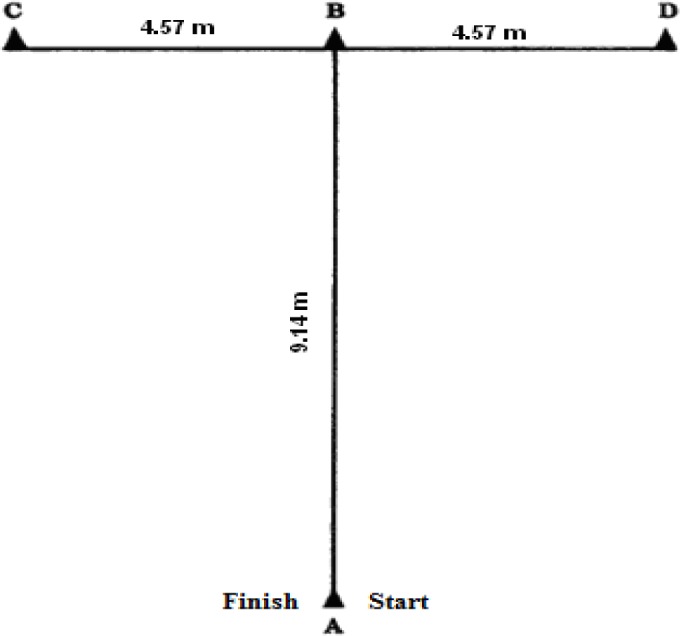
T Drill Agility Test

Four 22.86 cm collapsible agility cones were arranged as outlined in Semenick (1990) (Figure 1). At the tester’s signal, the subject sprinted forward 9.14 m and touched the tip of the cone (B) with their right hand. Then they performed a lateral shuffle to the left 4.57 m and touched the tip of the cone (C) with the left hand. Subjects then continued to shuffle 9.14 m to the right and touched the tip of the cone (D) with their right hand. They then shuffled 4.57 m to the left and touched point B with their left hand. Finally, subjects back-peddled 9.14 m passing through the finish at point A (Patterson et al., 2008). Time was measured using an electronic timing system (Prosport TMR ESC 2100, Tumer Engineering, Ankara,Turkey).

### Statistical Analyses

The mean and standard deviation values for each test were calculated for all players. A test for homogeneity of variance was applied to the data for each group for all comparisons and revealed no significant differences. An independent t-test was used to calculate comparisons according to division. One-way analysis of variance (ANOVA) was conducted to test for differences by playing position. If significant mean differences were found, Tukey post hoc analyses were used to determine the playing positions that showed significant differences.

## Results

Basketball players’ physical characteristics and test performances by division and playing positions are reported in Table 1 and 3.

Although first division players showed significantly better countermovement performance than second division players, second division players had significantly better 10m sprint performance (p≤0.05). Significant differences were not found between first and second division players in terms of body height, body mass, squat jump, VO_2_ max, 30 m sprint and T-drill agility measurements.

Basketball players’ isokinetic leg strength by division is reported in Table 2.

Statistically significant differences (p>0.05) were not found between first and second division players in terms of leg extensor and leg flexor strength.

In terms of player positions, forwards and centers were found to be significantly taller than guards (p≤0.05).

Centers were significantly heavier and their T-drill test performances were worse than both forwards and guards (p≤0.05). Guards were found to show significantly higher VO_2_max than centers (p≤0.05).

In contrast, Guards and forwards showed significantly better performance in the 10 and 30 m sprint tests than centers (p≤0.05). Statistically significant differences were not found between guards, forwards and centers in terms of PBF, countermovement jump or squat jump measurements.

The results of measurements of basketball players’ isokinetic leg strength by playing position are reported in Table 4.

Guards, forwards and centers had similar right leg extensor strength and left leg extensor strength at 60° × s^−1^ and 180° × s^−1^. Forwards and centers had significantly better left leg flexor strength than guards at 180° × s^−1^ (p≤0.05).

## Discussion

The purpose of the present study was to compare Turkish professional basketball players’ by division levels and playing position in terms of body composition, isokinetic leg strength, endurance, speed, vertical jump and agility. The main finding may provide coaches and athletes with information as to which physical attributes are specific to each playing position and therefore allow them to tailor fitness programs according to specific positions.

The physical characteristics of an athlete are important predictive factors of whether the athlete will reach the top level of their chosen sports discipline (Sallet et al., 2005). Sallet et al. (2005) did not find statistically significant differences between French first and second division basketball players in terms of physical characteristics. Schiltz et al. (2009) also demonstrated that the relative isokinetic and functional performances of professional basketball players were similar to those of junior players. Findings of the present study indicate that the physical characteristics and test performance of Turkish first division and second division players are statistically similar, except in countermovement jumps and 10m sprints. These results suggest that top level basketball players have similar physical characteristics.

A basketball player’s body height and body mass is one of the factors that determine court position (Drinkwater et al., 2008). In this study, guards were significantly shorter than both forwards and centers (p≤0.05). This finding echoes those of many other studies in the literature (Bradic et al., 2009; Ostojic et al., 2006; Sallet et al., 2005). Body composition is also an important aspect of fitness for team sports, as excess adipose tissue acts as dead mass in activities where body mass must be lifted repeatedly against gravity (Reilly et al., 2000). Indeed, in this study, centers had significantly greater body mass than both forwards and guards. This finding can be related to the fact that the performance of forwards and guards in terms of sprinting (10 and 30 m sprint test) and agility (T-drill test) was significantly superior to that of centers (p≤0.05).

Aerobic endurance in basketball is important for the player to maintain a high level of activity during the entire game, in both defense and offence (Ziv and Lidor, 2009). Abdelkrim et al. (2007) reported that during a basketball game guards cover a significantly higher distance and perform at higher intensity levels than centers and forwards. In accordance with Abdelkrim et al., the findings of the present study demonstrate that guards have higher VO_2_max values than centers and forwards (p≤0.05).

Basketball players’ leg power is an important feature for short-term and high intensity activities such as sprinting and jumping (Hoffman et al., 1996). The study findings demonstrate that first and second division players had similar leg extensor and leg flexor strength. In addition to this finding, guards, forwards, and centers show similar right and left leg extensor strength at 60° × s^−1^ and 180° × s^−1^, while forwards and centers had significantly better left leg flexor strength than guards at 180° × s^−1^. Schiltz et al. (2009) demonstrated better absolute isokinetic concentric performances for professional players than for junior players and those in the control group, and Bradic et al. (2009) revealed significant position differences in absolute isokinetic strength of the tested muscle groups. The greatest absolute concentric peak torque was produced by centers, followed by forwards and guards. Furthermore, Delextrat and Cohen (2009) showed a significant effect of a playing position on peak torques of the knee extensors as measured by the isokinetic dynamometer in women basketball players.

In conclusion, findings of the present study indicated that the physical performance of professional basketball players differ among guards, forwards and centers, while they do not differ significantly between first and second division players. According to the present study, particular court positions have different demands and specific physical attributes in professional basketball. Therefore, these results suggest that coaches should tailor fitness programs according to specific positions on the court.

## Figures and Tables

**Table 1:** Basketball players’ physical characteristics and test performances by division

	**First Division**	**Second Division**
Age (years)	24.0 ± 3.8	22.7 ± 4.0
Body Height (cm)	197.9 ± 8.0	195.7 ± 7.4
Body Mass (kg)	98.4 ± 12.3	94.7± 14.4
PBF (%)	10.9 ± 5.2	12 ± 3.5
10 m Sprint (s)	1.78 ± 0.8	1.72 ± 0.8*
30 m Sprint (s)	4.37 ± 0.21	4.35 ± 0.25
CMJ (cm)	40.6 ± 4.7	36.0 ± 5*
SJ (cm)	37.8 ± 5.7	34.7 ± 5.7
T-Drill Test (s)	9.49 ± 0.61	9.76 ± 0.57
Estimated VO_2_max (ml × kg^−1^ × min^−1^)	42.5 ± 8.6	44.5 ± 7.0

PBF : Percentage of body fat; CMJ: Countermovement Jump; SJ: Squat Jump; VO_2_max: Maximal oxygen uptake;

* p< 0.05

**Table 2:** Basketball players’ isokinetic leg strength by division

		
	**Leg Extensor**				**Leg Flexor**			
	
	60°Right (Nxm)	60°Left (Nxm)	180°Right ( Nxm)	180°Left (Nxm)	60°Right (Nxm)	60°Left (Nxm)	180°Right (Nxm)	180°Left (Nxm)
First Division	242.0 ± 56.4	247.7 ± 56.6	192.4 ± 40.6	189.9 ± 47.1	182.2 ± 37.8	174.7 ± 30.8	167.8 ± 34.3	151.5 ± 31.3
Second Division	250.4 ± 46.7	250.0 ± 43.6	181.5 ± 40.0	187.9 ± 34.3	178.3 ± 36.9	173.7 ± 31.2	160.2 ± 35.7	153.3 ± 32.5

**Table 3:** Basketball players’ physical characteristics and test performances by playing position

	Guards (n=14)	Forwards (n=15)	Centers (n=16)	All (n= 45)
Age (years)	22.9 ± 3.7	22.5 ± 3.9	24.5 ± 4.1	23.3 ± 3.9
Body Height (cm)	188.4 ± 5.4 #	196.9 ± 4.6	204.1 ± 2.5	196.8 ± 7.7
Body Mass (kg)	86.7 ± 9.4	91.7± 9.7	109.6± 8.1†	96.5 ± 13.4
PBF (%)	11.8 ± 3.0	9.4± 5.1	13.0 ± 4.4	11.4 ± 4.4
10 m Sprint (s)	1.72 ± 0.07	1.72 ± 0.07	1.8± 0.08†	1.75 ± 0.08
30 m Sprint (s)	4.25 ± 0.15	4.29 ± 0.19	4.48 ± 0.21†	4.34 ± 0.21
CMJ (cm)	38.2 ± 5.8	40.1 ± 5.1	36.6 ± 4.7	38.3 ± 5.3
SJ (cm)	36.4 ± 5.7	37.7 ± 5.2	34.7 ± 5.4	36.2 ± 5.5
T-Drill Test (s)	9.24 ± 0.56	9.48 ± 0.46	10.04 ± 0.35†	9.61 ± 0.57
VO_2_max (ml × kg^−1^ × min^−1^)	45.4 ± 8.3*	43.3 ± 7.2	42.1 ± 8.1	43.5 ± 7.8

# Significant difference from forwards and centers, p< 0.05

* Significant difference from centers, p< 0.05

† Significant difference from guards and forwards, p< 0.05

PBF : Percentage of body fat; CMJ: Countermovement Jump; SJ: Squat Jump; VO_2_max: Maximal Oxygen Uptake

**Table 4:** Basketball players’ isokinetic leg strength by playing position

		
	**Leg Extensor**				**Leg Flexor**			
	
	60°Right (Nxm)	60°Left (Nxm)	60°Right (Nxm)	60°Left (Nxm)	60°Right (Nxm)	60°Left (Nxm)	60°Right (Nxm)	60°Left (Nxm)
Guards	230.9 ± 45.3	238.3 ± 46.3	172.1 ± 38.0	178.1 ± 32.0	165.2 ± 30.7	161.7 ± 24.8	150.1 ± 25.3	139.1* ± 22.2
Forwards	246.1 ± 41.4	249.8 ± 41.2	190.5 ± 40.1	189.1 ± 31.7	180.1 ± 30.6	174.1 ± 27.9	162.5 ± 35.4	149.0 ± 28.9
Centers	261.2 ± 62.7	257.3 ± 60.4	196.5 ± 41.0	198.0 ± 53.0	194.1 ± 44.1	185.3 ± 35.0	177.9 ± 38.5	167.3 ± 36.1
All	246.4 ± 51.1	248.9 ± 49.8	186.7 ± 40.2	188.8 ± 40.6	180.1 ± 37.0	174.2 ± 3 0.7	163.8 ± 34.9	152.4 ± 31.5

* Significant difference from centers, p< 0.05
